# Body Physique, Body Composition, Physical Performance, Technical and Tactical Skills, Psychological Development, and Club Characteristics of Young Male Portuguese Soccer Players: The INEX Study

**DOI:** 10.3390/ijerph18073560

**Published:** 2021-03-30

**Authors:** Maryam Abarghoueinejad, Daniel Barreira, Cláudia Dias, Eduardo Guimarães, Adam D. G. Baxter-Jones, José Maia

**Affiliations:** 1 Centre of Research, Education, Innovation, and Intervention in Sport (CIFI2D), Faculty of Sport, University of Porto, 4200-450 Porto, Portugal; dbarreira@fade.up.pt (D.B.); cdias@fade.up.pt (C.D.); up201205873@edu.fade.up.pt (E.G.); jmaia@fade.up.pt (J.M.); 2 College of Kinesiology, University of Saskatchewan, Saskatoon, SK S7N 5B2, Canada; baxter.jones@usask.ca

**Keywords:** youth soccer, body physique and composition, performance, skills, motivation

## Abstract

Youth soccer performance is multifaceted, includes physical growth, biological maturation, and physical fitness, and is linked to the sporting environment to which the players are exposed. We aim to describe age-related associations in body physique, body composition, physical performance technical and tactical skills, psychological and club characteristics of male soccer players aged 12 to 14 years. A total of 157 male soccer players clustered into three age-cohorts (12, 13 and 14 years) were recruited from six soccer clubs. Anthropometric, body composition and body physique, biological maturation, physical performance, skill/game proficiency data, psychological characteristics, and clubs’ characteristics were collected. Group means were compared using analysis of variance and covariance. Fourteen years old players were significantly taller, heavier, leaner, faster, stronger, and technically more skilled than their younger peers (*p* < 0.05). Differences in physical performance and technical skills (*p* < 0.05) were found between age groups when adjusting for confounders of soccer training and biological maturation. No significant differences (*p* > 0.05) between age groups were found in psychological domains. Our findings suggest that age, biological maturation, and training volume are key factors influencing young soccer players’ performance and development. Further, clubs’ conditions provide players with ample resources for their success in training and competition.

## 1. Introduction

Youth soccer performance is multifaceted and is linked to physical growth, biological maturation, physical fitness, motor skills and psychological development, all entwined with the family and sporting environments. Interest in adolescent growth, biological maturation, and development, termed auxology, has a long history [1,2,3,4] and the young athlete, a specialized sub-group, has been extensively studied [5,6]. The Bronfenbrenner’s ecological systems theory indicates that a child’s development should be viewed as a complex system of relationships affected by multiple levels of the surrounding environment; from the family and school, to the cultural environment [7]. The young athletes’ physical growth, maturation, development, and performance are governed by the joint effects of their genetic make-up and the environment they develop within [8,9,10]. The young athletes surrounding environment is widely recognized as vital in the identification and optimization of young athletes’ performance, which in turn is linked to the continuous exposure to systematic training and competition.

In 2007, the Fédération Internationale de Football Association (FIFA) reported that 265 million people worldwide regularly played soccer, representing approximately 4.1% of the world’s total population, and that 38 million of these were registered as players with a soccer organization [11]. In addition to individual factors, performance is also linked to team factors [12] within multifaceted traits expressed in physical, physiological, and technical terms [13]. Fransen et al. [14], using a mixed-longitudinal study of Belgian soccer players (aged 5–20 years), reported segmented linear trajectories of physical performance change with age, reaching a plateau around 15–17 years-of-age. Valente-dos-Santos et al. [15] described, in 11–17 years-old Portuguese players, a positive association between biological maturation and training experience with physical performance, and Ford et al. [16] indicated that it was not only the quality, but also the quantity of time spent in soccer specific training that was positively related to the acquisition of skills and increased levels of physical performance. In contrast, Di Giminiani and Visca [17] showed that although soccer training improved the rate of physical performance in young Italian players, no links were identified between physical performance rates and changes with biological maturation. In a seven-year longitudinal study of Dutch players it was found that the knowledge of how to play the game increased not only with increasing age but was also dependant on field position [18]. Such overall findings have led to several organizations producing position statements. For example, the Canadian Dietitians and the American College of Sports Medicine position statements [19] endorse the fact that sports performance is directly mediated by an athletes’ body composition.

In addition to body physique, a young soccer player’s development is also linked with other factors such as psychological development, family support, coaches’ knowledge, and the club environment, to name but a few [20]. It has been found that elite youth soccer players performance levels are positively associated with higher task-orientated motivation, anticipation and decision-making [21], and the support of parents [22] and coaches [23].

To better understand young soccer players’ growth, maturation, development, and performance, within the various contexts of their training and competition, a holistic approach is required. Bronfenbrenner’s ecological systems theory [7] relies on a complex set of variables emerging from two main sources: the player (biological attributes, skill/game proficiency, and psychological characteristics), and the environment (key roles of family, coach, and club). In the present paper we describe age-associated differences in body physique, body composition, physical performance, technical and tactical skills psychological and club characteristics in male soccer players, aged 12–14 years of age. It was hypothesized that body physique, body composition, physical performance, technical and tactical skills, and psychological development and club characteristics would different between 12, 13 and 14 years old.

## 2. Materials and Methods

### 2.1. Setting

Soccer players were recruited as part of the *In Search of Excellence—A Mixed-longitudinal Study in Young Athletes* (INEX) study (2016–2019): https://inex-cifi2d.pt (accessed on 22 June 2020). The INEX study had three major aims: (1) to develop multivariate profiles of male youth athletes from 5 sports (soccer, basketball, handball, volleyball, and water-polo); (2) to model developmental trajectories, both within and between individuals, and to investigate predictors of success; and (3) to investigate the impact of competitive demands, with increasing age, on young athletes’ path to excellence.

The INEX study was designed, organized, and implemented by the Centre of Research, Education, Innovation, and Intervention in Sport (CIFI^2^D), located in the Faculty of Sport at the University of Porto (FADEUP), Portugal. The INEX study randomly recruited ~1000 young athletes (~200 participants per sport) and followed them consecutively over 3–4 years.

### 2.2. Quality Assurance

Since quality assurance is a key priority when planning a longitudinal study, a varied set of actions were implemented before initiating data collection, as well as during follow-up, in order to promote data integrity and validity [24]. First, the INEX study was led by a steering committee including senior researchers from Canada, Portugal, and the USA. The committee oversaw the design of the study, planned its settings for data collection, and developed all materials linked to the INEX study four main domains: biological, skill/game proficiency, psychological, and contextual. Second, these actions were led according to the most recent and important available literature, but also received inputs from both senior researchers and sport coaches. Third, the principal investigators trained all members of the assessment teams (one team per domain) to certify them. Fourth, a series of pilot studies were conducted to verify putative problems in real assessments as well as for feasibility, reliability control, data quality and data entry. Fifth, since missing data are presumed to occur at random in longitudinal or mixed-longitudinal designs, an analytical software to handle missing data will be used [25].

### 2.3. Study Design and Eligibility Criteria for Soccer Players

Six soccer clubs were recruited from the Porto Metropolitan area. They were selected due to their different levels of competitive experience as well as their commitment to participate in the INEX study. A mixed-longitudinal design with three age-cohorts (12, 13 and 14 years) was used. (Table 1). At baseline, a random sample of one hundred and fifty-seven male soccer players was recruited, cohort 1 (*n* = 49), cohort 2 (*n* = 51) and cohort 3 (*n* = 57) and this data is reported in the present paper. The data collection initiated in 2016. All assessments were performed in December. Written, informed consent was obtained from parents or legal guardians of each player, as well as their individual assent. The Ethics Committee (CEFADE 13.2017) approved the study, and the Porto Football Association gave formal permission for data collection.

### 2.4. Measurements

#### 2.4.1. Training Information

Players’ training experience, expressed as years of formal soccer training, was obtained from self-report questionnaires filled-out by players and cross-checked with registration histories, available from the official website of the Portuguese Football Federation (FPF): https://www.fpf.pt/Jogadores (accessed on 22 June 2020) [26]. A player registered for one competitive season in FPF indicates one year of formal soccer experience, for two competitive seasons, two years of experience, and so on. The weekly number, and the minutes, of the soccer-specific training sessions were also obtained from self-report questionnaires and completed by the managers/coordinators of the academies.

#### 2.4.2. Study Domains

The biological, skill/game proficiency, psychological, and contextual domains were grouped into two main clusters: (i) the young soccer players and (ii) their sporting environment (see Table 2).

##### Biological Domain

- Anthropometry

Anthropometric measurements were assessed with standard instrumentation and included height and sitting height measured with a wall mounted stadiometer (Holtain Ltd., Crymych, UK); either standing on the floor or seated on a sitting height table; bone widths measured with calipers (Siber Hegner GPM^®^, Zurich, Switzerland), girths with a anthropometric tape (Sanny^®^ Medical (SN-4010), São Paulo, Brazil), and skinfolds with a skinfold caliper (Holtain Ltd., Crymych, UK) following standard procedures [27]. Players were measured in their soccer kits with shoes removed.

- Body Composition

Body mass (kg) and fat mass (% from bioelectrical impedance) were measured using a valid and reliable [28] Tanita scale (Tanita^®^ model BC-418MA, Tokyo, Japan). Fat-free mass (kg) was derived according to the manufacturer’s formula. The following instructions were observed during all assessments: (1) Evening meals prior to testing should be taken as usual following players’ daily routines in terms of their nutritional habits; (2) Prior to arrival at the Lab players were asked to empty their bladder; (3) players were told to remain quietly positioned in the device as per the manufacturer instructions.

- Biological Maturation

A biological age—years from peak height velocity (PHV)—was estimated from anthropometrics using the Mirwald et al. [29] equation. Using measures of chronological age (years from birth), height, sitting height, body mass and their interactions the equation estimates the number of years the individual is from attainment of PHV, termed maturity offset. If maturity offset is positive (+) this indicates the number of years the subject is beyond the attainment of PHV, whereas a negative (–) maturity offset indicates the number of years the subject is prior to attainment of PHV. By subtracting maturity offset from chronological age, the age at peak height velocity can be estimated (age-at-PHV).

- Body Physique (Somatotype)

Somatotype components (endomorphy, mesomorphy, and ectomorphy) were derived from a set of 10 anthropometric measurements: height (cm), body mass (kg), biepicondylar humerus and femur widths (cm), arm flexed and calf girths (cm), triceps, subscapular, supraspinale, and medial calf skinfolds in mm, and body mass (kg) using the Health-Carter standard method [30].

- Physical Performance

Different components of physical performance were assessed using the following tests:

(1) high-intensity aerobic capacity was assessed using the Yo-Yo Intermittent Recovery Test—Level 1 (Yo-Yo IR1). Players performed repeated 2 × 20 m runs with an active recovery period of 10 s in between. The total distance covered (m) was used as the test result [31].
(2) lower limb speed movement was assessed using the foot tapping pedal test. Players performed the maximum number (reps) during 30 s in three trials and the best trial was used as the test result [32].
(3) running speed was assessed using the 5, 20 and 30 m sprint tests. Players ran in a straight line at full speed over 30 m. Time (s) was recorded using a photoelectric cells system, Speed Trap II (Brower Timing Systems LLC., Draper, UT, USA) with each pair of cells placed at each split (5, 20 and 30 m). Each player performed two trials and the best one was used as the test result [33].
(4) explosive strength was assessed using: (i) the squat jump and countermovement jump tests [34] using a AMTI OR6-WP force platform (Advanced Mechanical Technology Inc., Watertown, MA, USA) operating at 2000 Hz; jumping height (cm) was estimated; (ii) the standing long jump (cm) test [35]; and (iii) seated and standing 3 kg medicine ball throws (cm) [36]. Players performed three trails for each vertical jumping test and two trials for the standing long jump, and the best one was used as the test result. For the 3 kg seated and standing medicine ball throws, each player performed three trials and the mean was used as the test result.
(5) trunk strength and endurance were assessed using the sit-ups test. Players performed the maximum number (reps) of sit-ups during 60 s in two trials and the best trial was used as the test result [37].
(6) upper limb static strength was measured using the handgrip test. Players performed two maximal handgrip strength (kgf) with both hands using a hand-held dynamometer (Takei Digital Grip Strength Dynamometer Model T.K. K.5401, Takei Scientific Instruments Co., Ltd., Tokyo, Japan). The mean of best trails of both hands was used as the test result [37].
(7) agility was assessed using (i) the arrowhead test [38] and (ii) the T-test [39]. Time (s) was obtained using the photoelectric cells system Speed Trap II (Brower Timing Systems LLC., Draper, UT, USA). Players performed two trials and the best one was used as the test result.
(8) maximum ball velocity (m·s^−1^) during shooting was estimated from kicking speed test that determined using a stationary Doppler radar gun (Stalker ATS II) Players performed with their dominant leg three trails and the best trial was used as the test result [40].

##### Skill/Game Proficiency Domain

- Technical Skills

Soccer specific technical skills were assessed using the University of Queensland’s Football Skill Assessment Protocol [41], which comprises eight tests: (1) passing accuracy (points/kick) over 20 m; (2) lofted passing accuracy (points/kick) over 35 m; (3) shooting accuracy (points/kick) over 20 m; (4) performance during a wall-pass accuracy (points/kick) test; (5) maximum dribbling speed (m/s); (6) average juggling ability (number/min); (7) dynamic passing (cycles/min) test using two rebound boards set at 90 angle; and (8) dynamic passing (cycles/min) test using two rebound boards set at 135 angles.

- Tactical Skills

Tactical skills were assessed using the Tactical Skills Inventory for Sports (TACSIS) self-assessment questionnaire developed by Elferink-Gemser et al. [42] and culturally adapted to Portuguese population by Pereira et al. [43]. The TACSIS consists of 22 items and comprises (“knowing about ball actions” and “knowing about others”) and (“positioning and deciding” and “acting in changing situations”). All answers are on a 6-point Likert scale ranging from 1 (very poor) to 6 (excellent) or from 1 (almost never) to 6 (always).

- Match-Analysis

Match-analysis assessed behavioral events during competition, i.e., technical and tactical in medium-sided games (MSGs) 5 versus 5 with a goalkeeper adapting the field dimensions in 11 versus 11 to a 5 versus 5 configuration [44]. Further, the foot preference and lower limbs functional asymmetry index were obtained using the “System of assessment of functional asymmetry of the lower limbs in Football” (SAFALL-FOOT) [45]. Using global positioning systems (GPS) derived variables, the disposition and position of a soccer player in inter-individual and inter, intra-team coordination were assessed [46], and movement patterns applying heat maps. The type and intensity of the player’s movement frequency, duration in seven locomotor categories [47], known as time-motion analysis, were also measured. Players’ tactical processual performance was assessed with two tools: (1) the Tactical Assessment System (FUT-SAT) with its macro-categories (observation and outcomes), and seven categories will be used to understand the efficacy in performing the game principal, namely; defensive and offensive [48]; and (2) social networks will also be used to investigate the complexity of the interaction process established between competing players [49].

##### Psychological Domain

- Goal orientation

The goal orientation was assessed using the Task and Ego Orientation in Sport Questionnaire developed by Chi and Duda [50] and culturally validated for the Portuguese population by Fonseca [51]. Answers are on a 5-point Likert scale ranging from 1 (strongly disagree) to 5 (strongly agree). All players answered the questionnaire under the supervision of a trained member of the INEX research team.

- Motivational climate

The motivational climate was assessed using the Perceived Motivational Climate in Sport Questionnaire developed by Selfriz et al. [52] and culturally validated for the Portuguese population by Fonseca [53]. Answers are on a 5-point Likert scale ranging from 1 (strongly disagree) to 5 (strongly agree) that measures the players’ perceptions of the degree to which their respective team’s motivational climate is characterized by mastery and performance goals. All players answered the questionnaire under the supervision of a trained member of the INEX research team.

##### Contextual Domain

- Family structure

Information about parental support and family structure was obtained via a questionnaire [54] designed to assess the family size (parents and siblings), demographics (sex, age, and educational level) and sports’ history (past and current sports participation).

- Coach knowledge and competence

Coaches completed a questionnaire detailing: (1) demographics (age and sex); (2) academic degrees, sports’ history as a player and professional experience as a coach; (3) criteria for player’s selection based on performance factors and selection methodologies; and (4) information about the selection process, namely importance of selection indicators and criteria.

- Club information

Comprehensive information of different aspects of soccer clubs was assessed using questionnaire developed by members of INEX committee and research team. The questionnaire assessed sports’ infrastructures, equipment, human resources, communication, and developmental strategies linked to young players’ sporting trajectories in their clubs. All clubs’ presidents or directors completed the questionnaire under the supervision of a trained member of the INEX research team.

### 2.5. Statistical Analysis

Descriptive statistics are presented as means, standard deviations (±SD) and frequencies. ANOVA was used to compare means across the three age-cohorts and the Bonferroni’s test for post-hoc multiple comparisons; partial eta square (pη^2^)was used as a measure of effect size. ANCOVA was used to compare adjusted means accounting for weekly formal soccer training (combined from weekly number and minutes of formal soccer training) and biological maturation. All statistical analyses were performed using the IBM SPSS Statistics v.25 software and the significance level was set at 5%.

## 3. Results

The descriptive statistics, organized by age groups (body physique, body composition, physical performance, skill/game proficiency, and psychological domains), are presented in Table 3, Table 4 and Table 5, respectively.

### 3.1. Biological Domain

Fourteen years old soccer players were significantly (*p* < 0.001) taller, heavier, had greater fat-free mass, and were more mature than 12 years old and 13 years old (Table 3). Effect sizes ranged from pη^2^ = 0.237 for weight (kg) to pη^2^ = 0.587 for biological age (years from PHV). No significant differences (*p* > 0.05) were observed in fat mass nor for any of the somatotype components. A similar trend was observed in physical performance parameter (Table 4), with 14 years old showing significantly better results (*p* < 0.001) than their younger peers in almost all performance tests; effect sizes ranged from pη^2^ = 0.045 in foot tapping pedal test with preferred foot to pη^2^ = 0.380 in 20 m sprint. In sit-ups and foot tapping pedal test with non-preferred foot, no statistically significant differences (*p* > 0.05) were observed.

### 3.2. Skill Game Proficiency and Training Information Domain

When looking at technical skill tests (Table 4) it was found that, apart from 20 m shooting—accuracy (pts/kick), 14 years old players systematically outperformed their younger peers from the other two age-cohorts (*p* < 0.01) in all tests, with effect sizes ranging from pη^2^ = 0.07 in wall-pass to pη^2^ = 0.315 in pass rebound 90°. No statistically significant differences (*p* > 0.05) between age-groups were found in any of the tactical declarative skills sub-scales. However, older soccer players had more years of formal experience in soccer, accumulated more weekly sessions, and minutes of soccer specific training (14 years = 278 h versus 13 years = 249 and 12 years = 209 h).

### 3.3. Psychological Domain

The data for the psychological domain (goal orientation and motivational climate) are presented in Table 5. No statistically significant differences (*p* > 0.05) were found for any of the sub-scales among players of the three age-cohorts.

### 3.4. Controlling for Biological Maturation

Table 6 shows the ANCOVA results when controlling for biological maturation and weekly formal soccer training. Significant differences (*p* < 0.05) between 14 years old players and 13 years old in the physical performance set remained, except in tapping pedal test with preferred foot, handgrip, medicine ball throw, and 5 m sprint among the three age-cohorts (*p* > 0.05). Also, differences between age-cohorts in technical skills set only remained in dribbling and pass rebound 90° favoring 14 years old.

### 3.5. Contextual Domain

Table 7 shows clubs’ characteristics revealing a substantial variability among them, namely in the number of sports available, in the number of soccer players as well as in the number of the clubs’ soccer section. All clubs had their own facilities, video room, and warm-up area, but none had hydrotherapy. Most clubs had synthetic grass and four of them had natural grass fields. Almost all clubs had a physician, nutritionist, physiotherapist, and a psychologist. Clubs had mostly coached with Union of European Football Associations (UEFA) C (level 1) certification, and all used social media.

## 4. Discussion

The current study investigates soccer players age associated differences in body physique, body composition, physical performance. Technical and tactical skills psychological and club characteristics. Fourteen years old soccer players were more mature, taller, heavier and had less fat mass and greater lean mass than 12 and 13 years old. Fourteen years old were also less endomorphic and more mesomorphic. The oldest age group also and better physical performance and technical skills but not improved tactical skills. The significant differences between age groups for physical performance and technical skills remained in some of the measures when the confounders of maturity status and training were controlled. No significant differences were found for any of the psychological characteristics assessed or club characteristics.

It is well-known that young players’ development is multidimensional and complex, however the challenge is to assess and integrate results appropriately [8]. It is also important that a more encompassing understanding of young athletes’ pathways to success incorporates the relationships of performance to environmental exposures [55,56]. This is a daunting task that requires a coherent and encompassing holistic framework [57,58]. The present paper identifies some of the challenges and aims to fill this gap using Bronfenbrenner bio-ecological model [7].

The present cohort of soccer players started their formal soccer training during childhood, and we concur with Ford et al. [59] that early exposure to organized systematic practice may expedite developmental attainments in physical performance and technical skills. Furthermore, we found that weekly soccer specific training sessions increased from three, at 12 years of age, to four sessions for older players. This led to a greater accumulation of minutes per week of soccer training and these results are consistent with Ford et al. [60] data. It has been predicted that conducting adequate training in these windows of opportunity will accelerate and enhance the development of youth performance capacities [56].

### 4.1. Biological Domain

Our results showed that soccer players’ physical, performance and skill characteristics differ between age groupings, favoring the older and more mature players. A similar trend was also reported in young soccer players by Slimani and Nikolaidis [61] systematic review, and is supported by empirical research in athletic youth from individual and team sports [62], as well as in other studies of young soccer players [14,63,64]. During the adolescent growth spurt, age-differences in physical performance become pronounced [65] due to dissimilarities among players’ timing and tempo of their statural growth. Furthermore, increases in muscle mass, governed by a complex hormonal cascade affecting aerobic and anaerobic enzyme systems tend to favor older youth [5].

After controlling for differences in biological maturation and soccer training, many previous un-controlled physical performance differences became non-significant between 12 and 14 years of age. This result is in line with other research emanating from other cohorts of Portuguese soccer players [15]. We contend that different training foci across age categories [66] associated with advancing biological maturation moderated the affects [16] which maximized young players’ training responses [17] and prepared them for competition [56]. However, this was not true for all tests of performance. In the Yo-Yo IR1, speed (20 and 30 m sprint), jumps and agility tests differences still favored 14 years-old relative to 13 years-old, after adjusting for biological maturation and weekly training. This may be due to increased specificities of training demands, differences in lower-limb lengths favoring 14-year-olds as well as increased neuromuscular coordination in the older age group [67].

### 4.2. Skill Game Proficiency Domain

In line with previous reviews [11,68], we found that older players outperformed their younger peers in soccer-specific skills and support previous results [15,64]. Young soccer players systematically improve from 12 to 14 year of age in all technical skills tests, except in the shooting accuracy. This improvement can be explained in part by the combined effects of organized practice [69], and by the process of normal growth [70]. However, after adjusting for biological maturation and weekly formal soccer training, significant differences disappeared between age-cohorts apart from dribbling and rebound pass 90° test. It can be speculated that 14-year-olds advantages may be due to their increased coordinative abilities, for example spatial orientation and kinesthetic differentiation [71]. Furthermore, since dribbling is one of the most performed techniques during match play, followed by passing [72] and 14-year-old players have played and practices for longer, this could explain these results [73].

In contrast with Kannekens et al. [18], who assessed tactical declarative skills using the TACSIS in elite youth soccer players, we found no significant differences across age-groups, despite the relatively high scores. It is important to emphasize that tactical skills were assessed with a self-reporting inventory that evaluates perceptual-cognitive skills and are linked to knowledge about rules and goals of the game associated to perception of response nomination (i.e., “knowing what to do”) which reflects players’ own perceptions competence in soccer [74]. Our findings probably reflect players’ narrowed age distribution (12–14 years old), with their putative limited exposure to systematic competitive events, and/or their willingness to cope with socially desirable answers.

### 4.3. Psychological Domain

In the psychological domain, results from both goal orientation and motivational climate showed no statistically significant differences between age-cohorts. Nevertheless, our results are in line with a study highlighting that young soccer players experience their goal orientation mostly as task orientation [75]. This suggest that our soccer players tended to be self-focused and target improvement may also adopt personal development strategies as well as learning new skills as criteria for competence. Indeed, Nicholls [76] described achievement goal orientations as internalizations of the contextual achievement cues. Additionally, our data also suggests that players perceived the motivational climate as task climate. As Ames [77] argued, there is a socialization influence on young people’s achievement goal orientations, and exposure to a strong motivational climate can influence the salience and adoption of the related achievement goal orientation. Therefore, the findings of present study may indicate a putative positive effect of players’ contexts (e.g., training environment perceptions and coaches’ expectations) to elevate their levels of task achievements [23,78,79].

### 4.4. Contextual Domain

According to the ecological approach [7], researchers need to consider the environment in which young soccer players are embedded to be able to better understand the complexities of their development [80]. The results of this study draw some parallels with previous data [81], i.e., we reported that the INEX enlisted soccer clubs represent local grassroots clubs and can be considered as critical elements in also scouting and recruitment. Furthermore, they offered an environment focusing on providing players with resources both on and of the pitch, using systematic methodologies that may accelerate and maximize youngsters’ soccer potentials and smooth their career transitions [82].

This study is not without limitations. First, our sample is small, but similar to estimates given by G*Power (effect size = 0.25, α = 0.05, power = 0.80, number of groups = 3: ANOVA, fixed effects, omnibus, one-way); further, it is not representative in terms of players and clubs of the Portuguese youth soccer population. It is recommended that care be taken when generalizing the present results. Second, we only focused on a set of variables that may be more appealing to Human Biologists like growth, biological maturation, motor performance, perceived knowledge, and psychological factors. However, the overall INEX study also has several strengths: (1) it is framed within Bronfenbrenner’s bio-ecological theory on human development; (2) considers a wide range of variables evolving from two main sources—the player orbit (biological, skill/game proficiency, psychological) as well as its contexts (family, coach, club); (3) has a series of coherent procedures to guarantee the quality of data acquisition during the four years; and (4) its steering committee comprises well-known experts in the field that are “safe guards” of the overall importance of the study.

### 4.5. Implications for Human Biology

The interest of Human Biologists in studying the various implications of children and adolescents’ normal and abnormal growth, physical performance and development within a variety set of conditions and contexts are always with us in a rapid changing world. Furthermore, young athletes, as a special group from any population, also require their utmost attention given: (1) their constant exposure to the manifold demands of training regimens and competitions; (2) physiological and psychological adaptations as well as increased likelihood of injuries; (3) coaches, parental and societal expectations for success. In summary, this research project, with its future aims to describe longitudinal trajectories of development has promises for a broader understanding on the complex dynamical relationships in young soccer players’ growth, body composition and body shape, biological maturation, physical performance, and soccer-specific skills within the network of influences from their varied contexts (family, coaches, and clubs).

## 5. Conclusions

In conclusion, 14 years-old players were found to be more advanced in body physique and had body compositions that were in line with their advanced biological maturation. Furthermore, the 14 years-old outperformed their younger peers in all physical performance and tactical skills components. These components were related to both advanced maturity and increased training. Young soccer players’ tactical skills as well as psychological characteristics (goal orientation and motivational climate) did not differ across age-cohorts. Finally, clubs offer a variety of conditions aiming to enhance players success in their response to training and competition.

## Figures and Tables

**Table 1:** Chronological age across cohorts, number of players per cohort (at baseline) and years overlap.

Cohorts	Chronological Age					
Cohort 1	12 (*n* = 49)	13	14	15		
Cohort 2		13 (*n* = 51)	14	15	16	
Cohort 3			14 (*n* = 57)	15	16	17

**Table 2:** Measures, scales, and variables of players’ and clubs’ domains.

Domain	Measure/Scale	Variables	Participation
**Biological domain**			
Anthropometry	Standard protocol (ISAK)	Height Sitting height Girths Skinfolds	All players
Body composition	Bioimpedance scale (Tanita BC-418MA)	Weight Fat percentage Fat-free mass BMI	All players
Biological maturation	Standard protocol	Maturity offset	All players
Somatotype	Heath-Carter method	Body physique	All players
Physical performance	Physical fitness tests	Yo-Yo IR1 Foot tapping pedal test 5, 20 and 30 m sprints Squat jump Countermovement jump Standing long jump Seated medicine ball throw Standing medicine ball throw Sit-ups Handgrip Arrowhead test T-test Ball velocity	All players
**Skill/game proficiency domain**			
Technical skills	University of Queensland Football Skill Assessment Protocol	Passing accuracy over 20 m Lofted passing accuracy over 35 m Shooting accuracy over 20 m Wall-pass accuracy test Dribbling speed Juggling ability Passing rebound boards at 90° Passing rebound boards at 135°	All players
Tactical skills	TACSIS	Positioning and decisioning Knowing about ball actions Knowing about others Acting in changing situation	All players
Match-analysis	SAFALL-FOOT	Lower limb functional asymmetry	All players
	Global Positioning System (GPS)	Inter-individual coordination Inter, intra-team coordination Movement pattern (heat maps)	All players
	FUT-SAT	Offensive tactical performance index Defensive tactical performance index	All players
	Social networks	Passing networks	All players
Training information	FPF history registration Training history questionnaire	Soccer-specific years of training Soccer-specific weekly session Soccer-specific weekly minutes per session	All players
**Psychological domain**			
Goal orientation	PMCSQ scale	Task Ego	All players
Motivational climate	TEOSQ scale	Performance climate Task climate Emphasis on mistakes climate	All players
**Contextual domain**			
Family structure	Questionnaire	Family size Demographics Family sports’ history	All parents
Coach knowledge and competence	Questionnaire	Demographics Academic degrees and sports’ history Indicators and criteria for player’s selection	All coaches
Club information	Questionnaire	Characteristics Infrastructures Transportation availability Human resources Communication	All clubs

**Table 3:** Descriptive statistics [means and standard deviations (SD)], ANOVA results (F and *post hoc* tests), and partial eta square (pη^2^) for the biological domain.

Biological Domain	12 Years (*n* = 49)	13 Years (*n* = 51)	14 Years (*n* = 57)	F	*Post Hoc*	pη^2^
Variables	Mean ± SD	Mean ± SD	Mean ± SD			
Decimal age (years)	12.49 ± 0.32	13.56 ± 0.25	14.47 ± 0.27	632.35 ***	C3 > C2 > C1	0.891
*Anthropometry*						
Height (cm)	155.21 ± 8.05	162.66 ± 8.68	168.00 ± 7.67	35.50 ***	C3 > C2 > C1	0.316
Sitting height (cm)	80.98 ± 3.96	84.56 ± 4.72	87.82 ± 4.54	31.13 ***	C3 > C2 > C1	0.289
Body composition						
Weight (kg)	44.70 ± 8.99	51.78 ± 10.15	57.76 ± 9.84	23.93 ***	C3 > C2 > C1	0.237
Fat mass (%)	18.01 ± 3.54	17.31 ± 4.10	17.09 ± 3.15	0.919 ^ns^	-	0.012
Fat-free mass (kg)	36.92 ± 6.41	42.57 ± 7.14	47.67 ± 6.58	33.73 ***	C3 > C2 > C1	0.305
*Biological maturation*						
Maturity offset (years-to-PHV)	−1.18 ± 0.59	−0.19 ± 0.68	0.70 ±0.68	108.7 ***	C3 > C2 > C1	0.587
*Somatotype*						
Endomorphy	3.4 ± 1.7	2.9 ± 1.3	2.7 ± 1.1	2.67 ^ns^	-	0.034
Mesomorphy	3.8 ± 0.9	3.5 ± 1.1	3.7 ± 1.0	1.08 ^ns^	-	0.014
Ectomorphy	3.5 ± 1.2	3.5 ± 1.3	3.4 ± 1.6	0.07 ^ns^	-	0.001
*Physical performance*						
Yo-Yo IR1 (m)	369.00 ± 154.9	533.60 ± 214.0	715.74 ± 276.27	26.17 ***	C3 > C2 > C1	0.281
Foot tapping pedal (pf) (reps)	29.69 ± 4.88	30.70 ± 6.86	32.63 ± 5.33	3.56 *	C3 > C1	0.045
Foot tapping pedal (nf) (reps)	22.63 ± 9.34	24.24 ± 11.55	25.41 ± 10.00	0.94 ^ns^	-	0.012
5 m sprint (s)	1.40 ± 0.89	1.37 ± 0.10	1.28 ± 0.97	21.16 ***	C3 > C2, C3 > C1	0.218
20 m sprint (s)	3.88 ± 0.20	3.80 ± 0.20	3.52 ± 0.19	46.49 ***	C3 > C2, C3 > C1	0.380
30 m sprint (s)	5.43 ± 0.32	5.28 ± 0.29	4.90 ± 0.28	44.44 ***	C3 > C2, C3 > C1	0.369
Countermovement jump (cm)	24.23 ±4.36	25.62 ± 3.78	29.36 ± 4.27	21.98 ***	C3 > C2, C3 > C1	0.222
Standing long jump (cm)	172.71 ± 21.90	177.06 ± 21.17	196.22 ± 18.90	19.68 ***	C3 > C2, C3 > C1	0.205
Medicine ball throw (cm)	426.02 ± 98.64	494.86 ± 110.09	605.73 ± 100.89	40.71 ***	C3 > C2 > C1	0.347
Sit-ups (reps)	22.55 ± 4.60	21.75 ± 5.16	23.30 ± 5.04	1.32 ^ns^	-	0.269
Handgrip (kgf)	22.35 ± 4.67	27.08 ± 6.21	31.92 ± 5.63	38.82 ***	C3 > C2 > C1	0.337
T-test (s)	10.52 ± 0.65	10.35 ± 0.63	9.82 ± 0.53	19.12 ***	C3 > C2, C3 > C1	0.201

*** *p* < 0.001; * *p* < 0.05; ^ns^ = non-significant; pf = preferred foot; nf = non-preferred foot.

**Table 4:** Descriptive statistics [means and standard deviations (SD)], ANOVA results (F and *post hoc* tests), and partial eta square (pη^2^) for the skill/game proficiency domain.

Skill/Game Proficiency Domain	12 Years (*n* = 49)	13 Years (*n* = 51)	14 Years (*n* = 57)	F	*Post Hoc*	pη^2^
Variables	Mean ± SD	Mean ± SD	Mean ± SD			
*Technical skills*						
Passing accuracy 20 m (pts/kick)	3.67 ± 1.08	3.54 ± 0.99	4.27 ± 1.30	6.28 **	C3 > C2, C3 > C1	0.075
Lofted passing 35 m (pts/kick)	2.61 ± 1.25	3.47 ± 1.66	4.89 ± 1.70	29.12 ***	C3 > C2 > C1	0.274
Shooting accuracy 20 m (pts/kick)	2.80 ± 1.01	3.00 ± 1.10	3.01 ± 1.19	0.587 ^ns^	-	0.008
Wall-pass accuracy (pts/kick)	7.30 ± 3.40	9.14 ± 4.07	10.49 ± 4.24	8.62 ***	C3 > C1	0.101
Dribbling speed (m/s)	2.20 ± 0.19	2.33 ± 0.18	2.47 ± 0.16	28.26 ***	C3 > C2 > C1	0.270
Juggling ability (reps/min)	16.08 ± 11.62	22.61 ± 16.40	37.65 ± 19.22	17.00 ***	C3 > C2, C3 > C1	0.143
Pass rebound 90° (cycles/min)	0.58 ± 0.06	0.61 ± 0.05	0.68 ± 0.06	35.48 ***	C3 > C2, C3 > C1	0.315
Pass rebound 135° (cycles/min)	0.57 ± 0.06	0.60 ± 0.06	0.65 ± 0.05	22.60 **	C3 > C2, C3 > C1	0.227
*Tactical skills*						
Positioning and decisioning	4.51 ± 0.61	4.51 ± 0.66	4.67 ± 0.69	1.03 ^ns^	-	0.013
Knowledge about ball action	4.57 ± 0.68	4.67 ± 0.62	4.53 ± 0.75	0.60 ^ns^	-	0.008
Knowing about others	4.46 ± 0.62	4.55 ± 0.65	4.53 ± 0.73	0.25 ^ns^	-	0.003
Action in changing situations	4.51 ± 0.69	4.47 ± 0.86	4.60 ± 0.77	0.46 ^ns^	-	0.003
*Training information*						
Years of training (years)	4.33 ± 1.41	4.51 ± 1.30	5.68 ± 1.42	15.15 ***	C3 > C2, C3 > C1	0.165
Weekly session (n)	3.00 ± 0.00	3.18 ± 0.38	3.37 ± 0.48	13.38 ***	C3 > C2, C3 > C1	0.148
Weekly minutes	208.8 ± 25.77	248.82 ± 40.60	277.90 ± 44.83	42.82 ***	C3 > C2 > C1	0.357

*** *p* < 0.001; ** *p* < 0.01; ^ns^ = non-significant.

**Table 5:** Descriptive statistics [means and standard deviations (SD)], ANOVA results (F and *post hoc* tests), and partial eta square (pη^2^) for the psychological domain.

Psychological Domain	12 Years (*n* = 49)	13 Years (*n* = 51)	14 Years (*n* = 57)	F	*Post Hoc*	pη^2^
Variables	Mean ± SD	Mean ± SD	Mean ± SD			
*Goal orientation*						
Task	4.29 ± 0.65	4.50 ± 0.40	4.31 ± 0.48	1.35 ^ns^	-	0.017
Ego	2.40 ± 0.90	2.37 ± 0.93	2.64 ± 0.99	1.31 ^ns^	-	0.017
*Motivational climate*						
Performance climate	2.69 ± 0.71	2.65 ± 0.71	2.82 ± 0.63	0.962 ^ns^	-	0.012
Task climate	4.45 ± 0.59	4.50 ± 0.43	4.35 ± 0.40	0.350 ^ns^	-	0.005
Emphasis on mistakes climate	3.16 ± 0.87	3.43 ± 1.02	3.24 ± 0.93	1.062 ^ns^	-	0.014

^ns^ = non-significant.

**Table 6:** Descriptive statistics [adjusted means and standard error (S.E.)] from ANCOVA (covariates: weekly formal soccer training sessions, and biological maturation) of young soccer players.

	12 Years (*n* = 49)	13 Years (*n* = 51)	14 Years (*n* = 57)	F	*Post Hoc*	pη^2^
Variables	Mean ± SD	Mean ± SD	Mean ± SD			
*Physical performance*						
Yo-Yo IR1 (m)	427.14 ± 47.04	520.14 ± 30.77	667.54 ± 41.05	5.81 *	C3 < C2, C2 < C1	0.082
20 m sprint (s)	3.76 ± 0.03	3.80 ± 0.02	3.63 ± 0.03	8.52 ***	C3 < C2	0.102
30 m sprint (s)	5.22 ± 0.05	5.29 ± 0.04	5.09 ± 0.05	5.45 **	C3 < C2	0.068
Countermovement jump (cm)	25.78 ± 0.82	25.56 ± 0.57	28.10 ± 0.74	3.72 *	C3 > C2	0.047
Standing long jump (cm)	181.86 ± 4.10	176.55 ± 2.81	187.81 ± 3.7	3.13 *	C3 > C2	0.046
T-Test (s)	10.39 ± 0.12	10.36 ± 0.08	9.96 ± 0.11	4.05 *	C3 > C2	0.052
*Technical skills*						
Dribbling speed (m/s)	2.26 ± 0.34	2.32 ± 0.23	2.43 ± 0.30	4.96 **	C3 > C1	0.062
Pass rebound 90° (cycles/min)	0.58 ± 0.01	0.61 ± 0.09	0.67 ± 0.11	10.08 ***	C3 > C2, C3 > C1	0.118

*** *p* < 0.001; ** *p* < 0.01; * *p* < 0.05.

**Table 7:** Descriptive statistics for clubs’ data.

Clubs (*n* = 16)	Mean ± SD	Min-Max	n (%)
*Club characteristics*			
Number of sports within the club	2.00 ± 0.63	1-3	
Number of soccer players	487.83 ± 139.99	325-700	
Number of years of the club’s soccer section	72.70 ± 32.71	14-108	
*Club infrastructure*			
Own facilities (Yes/No)			6(100)/0(0.00)
Complementary equipment			
Gym (Yes/No)			5(83.3)/1(16.7)
Warm-up area (Yes/No)			6(100)/0(0.00)
Medical/Physiotherapy office (Yes/No)			6(100)/0(0.00)
Hydrotherapy (Yes/No)			0(0.00)/6(100)
Video Room (Yes/No)			6(100)/0(0.00)
Wooden/parquet flooring (Yes/No)			2(33.3)/4(66.7)
Synthetic grass (Yes/No)			5(83.3)/1(16.7)
Natural grass field (Yes/No)			4(66.7)/2(33.3)
*Transportation availability*			5(83.3)/1(16.7)
Bus			5(83.3)/1(16.7)
Metro			4(66.7)/2(33.3)
*Human resources*			
Number of coaches	35.17 ± 13.36	20-58	
*Coaches’ level category certification*			
Number of coaches with level 1	35.17 ± 13.36	7-36	
Number of coaches with level 2	4.33 ± 1.50	3-7	
Number of coaches with level 3	1.00 ± 0.00	1-1	
Number of coaches with level 4	1.00 ± 0.81	0-2	
*Staff*			
Physician (Yes/No)		1-1	6(100)/0(0.00)
Psychologist (Yes/No)		0-1	5(83.3)/1(16.7)
Physiotherapist (Yes/No)		1-5	6(100)/0(0.00)
Nutritionist (Yes/No)		0-1	6(100)/0(0.00)
Nurse (Yes/No)		0-1	1(16.7)/5(83.3)
*Club communication*			
Social media (Yes/No)			6(100)/0(0.00)

## Data Availability

Data are available upon request due to ethical restrictions. Individuals or readers interest in the data should contact José Maia (jmaia@fade.up.pt).
